# Transcriptional Expressions of CXCL9/10/12/13 as Prognosis Factors in Breast Cancer

**DOI:** 10.1155/2020/4270957

**Published:** 2020-09-09

**Authors:** Yan Li, Mingqiang Liang, Yuxiang Lin, Jinxing Lv, Minyan Chen, Peng Zhou, Fangmeng Fu, Chuan Wang

**Affiliations:** ^1^Breast Surgery Ward, Department of General Surgery, Fujian Medical University Union Hospital, Fuzhou, Fujian Province 350001, China; ^2^Department of Thoracic Surgery, Fujian Medical University Union Hospital, Fuzhou, Fujian Province 350001, China

## Abstract

CXCLs play critical roles in antitumor immunity by activating tumor-specific immune responses and stimulating tumor proliferation, thus affecting patient outcomes. However, the expression and prognostic values of CXCLs in breast cancer have not been well clarified. The aim of this study was to investigate the impact of CXCLs transcriptional expression on breast cancer patients. Oncomine database, GEPIA (Gene Expression Profiling Interactive Analysis), UALCAN, Kaplan–Meier Plotter, TIMER (Tumor Immune Estimation Resource), and DAVID were used in our study. The transcriptional levels of CXCL9/10/11/13 in breast cancer tissues were significantly elevated while the transcriptional levels of CXCL1/2/3/12 were decreased based on intersections of Oncomine database and GEPIA. Among them, breast cancer patients with high transcriptional levels of CXCL2/9/10/12/13 and low transcriptional level of CXCL3 were associated with a better prognosis. We also found that most of CXCLs expressions are significantly correlated with known prognostic factors, such as patient's age, major subclasses, individual cancer stages, and nodal metastasis status. In addition, the expression of CXCL9/10/12/13 was also indicated to be correlated with the infiltration of six types of immune cells (B cells, CD8+ T cells, CD4+ T cells, macrophages, neutrophils, and dendritic cells). The functions of differentially expressed CXCLs are primarily related to the immune response and cytokine-cytokine receptor interactions. Our results may provide novel evidence of new prognostic or predictive biomarkers for breast cancer patients.

## 1. Introduction

CXCLs, in which C stands for cysteine and X represents any amino acid, are a family of structurally similar inflammatory chemokines. By binding to cognate CXCRs, these CXCLs play indispensable roles in various biological processes, including angiogenic, angiostatic, tumor initiation, promotion, and progression [1–3]. CXCLs chemokine family were separated into two structurally distinct groups, ELR^+^ and ELR^−^, based on the presence or absence of a Glu-Leu-Arg motif at the N-terminus, which appears to be important in ligand/receptor interactions on evoking immune cells to eliminate tumor cells. The ELR^+^ members cover CXCL1 to CXCL3, CXCL5 to CXCL8, and CXCL17 while the ELR^−^ members include CXCL4, CXCL9 to CXCL13, and CXCL16. The latest study has revealed that CXCL family was vital to regulate tumor progression by interaction between tumor and the tumor microenvironment in pancreatic cancer [4]. As to renal cell carcinoma, low transcriptional levels of CXCL1/2/3/5/13 were associated with a significantly better prognosis [5]. Conversely, CXCL1 and CXCL2 were found upregulated in colorectal cancer, which play an important role in treatment resistance and metastasis [6]. Therefore, we surmised that CXCLs could be utilized as potential therapeutic targets and prognostic biomarkers for neoplasm.

Breast cancer is the most common malignant tumor and the leading cause of cancer deaths among women worldwide, accounting for 24.2% of newly diagnosed cancers and 15.2% of mortality [7]. Approximately 5–10% of female breast cancer patients might have metastatic disease at presentation and 13% of women with primary stage IV breast cancer survive 10 years after diagnosis [8]. Breast cancer is a highly heterogeneous disease and may be divided into three major subtypes according to different molecular features as follows: luminal group, basal-like group, and HER-2 overexpressing group [9]. Although some progress has been made in identifying the therapeutic targets and prognostic biomarkers, some biomarkers involved in tumor microenvironment may uncover new possible avenues to individualized treatment.

To date, several studies have demonstrated the expression profile and function of some CXCLs in breast cancer. For example, silencing CXCL7 could significantly reduce invasive and metastatic properties of breast cancer stem cells, thus leading to an improved outcome [10]. CXCL10 plays indispensable role in breast cancer patients for immune checkpoint-based therapies [11], while CXCL1 had been shown to be involved in resistance to cancer chemotherapies [12]. However, the role of distinct CXCLs family members remained unknown in the development and progression of breast cancer. We performed this study by analyzing the expression patterns and potential functions of different CXCLs family members in patients with breast cancer based on some public databases, thus mining some therapeutic targets and prognostic biomarkers.

## 2. Materials and Methods

### 2.1. Oncomine Database

Oncomine database (http://www.oncomine.org) is an online cancer microarray database and synthetic gene-wide data-mining platform [13]. We compared the transcriptional levels of 15 CXCLs members in different cancer tissues with their corresponding adjacent normal controls from Oncomine database, using Student's *t*-test to generate a *p* value. The cutoffs of *p* value and fold change were defined as 0.05 and 2, respectively.

### 2.2. Gene Expression Profiling Interactive Analysis

The Gene Expression Profiling Interactive Analysis (GEPIA) (http://gepia.cancer-pku.cn/) integrates tremendous amount of tumor and non-tumor samples from The Cancer Genome Atlas (TCGA) and the Genotype-Tissue Expression (GTEx) database, providing differential expression analysis, correlation analysis, and patient survival analysis online [14]. In our study, GEPIA was used to analyze the expression of CXCLs in breast tumors with corresponding breast tissues. Cutoff of *p* value was 0.05 and Log 2 FC was 1 (fold change was 2). We also obtained the top 100 similar expression protein‐coding genes with certain CXCLs family members in the “TCGA Tumor”—BRCA Tumor dataset by similar genes module for Gene Ontology (GO), Kyoto Encyclopedia of Genes and Genomes (KEGG) analysis.

### 2.3. UALCAN

UALCAN (http://ualcan.path.uab.edu) is a comprehensive web resource based on TCGA database level 3 RNA-seq and clinical data from 31 cancer types [15]. It can be used to estimate relative transcriptional expression of query gene between tumor and normal samples as well as relative clinicopathologic parameters on patient survival. In this study, UALCAN was used to analyze the association between mRNA expressions of 15 CXCLs family members in breast invasive carcinoma and known prognostic factors (patient's age, individual cancer stages, major subclasses, and nodal metastasis status).

### 2.4. Kaplan–Meier Plotter Analysis

The association of CXCLs expression with overall survival (OS) of patients with breast cancers was analyzed by Kaplan–Meier Plotter (http://www.kmplot.com), which is able to assess the effect of 54k genes on survival in 21 cancer types [16]. Patients with breast cancer were divided into high and low expression group by best cutoff. The number-at-risk cases, HRs, and log rank *p* values can be found at the Kaplan–Meier Plotter web page. Relapse-free survival (RFS) of ER- (estrogen receptor-) positive group and ER-negative group was furthermore assessed. Statistically significant difference was considered when a log rank *p* value <0.05.

### 2.5. Tumor Immune Estimation Resource Database Analysis

Tumor Immune Estimation Resource (TIMER) (https://cistrome.shinyapps.io/timer/) acts as a web server for systematical analysis of tumor-infiltrating immune cells across 32 tumor types from TCGA [17]. Gene module was used to explore correlation between CXCLs expression and abundance of immune infiltrates, including tumor purity, B cells, CD8+ T cells, CD4+ T cells, macrophages, neutrophils, and dendritic cells (DCs) by Spearman's correlation. Cancer types are listed as BRCA (breast invasive carcinoma), BRCA-Basal, BRCA-Her2, and BRCA-Luminal. The expression levels of CXCLs are log 2 RSEM.

### 2.6. Gene Ontology (GO) and Kyoto Encyclopedia of Genes and Genomes (KEGG) Analysis

DAVID (https://david.ncifcrf.gov/home.jsp) is an integrated database that helps investigators better understand the biological meaning behind a large list of genes. GO and KEGG analysis were conducted by DAVID and visualized with R project using a “ggplot2” package [18, 19]. Biological processes, cellular components, and molecular functions were included in the GO enrichment analysis, while KEGG analysis defined the pathways related to the CXCLs.

## 3. Results

### 3.1. Transcription Levels of CXCLs in Patients with Breast Cancer

**Figure 1:**
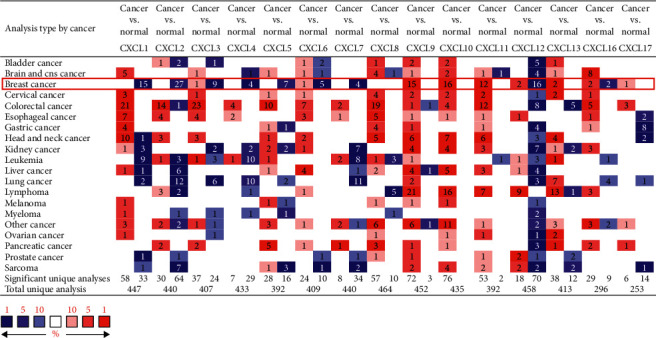
Transcriptional expression of CXCLs in 20 different types of cancers (Oncomine database).

**Figure 2:**
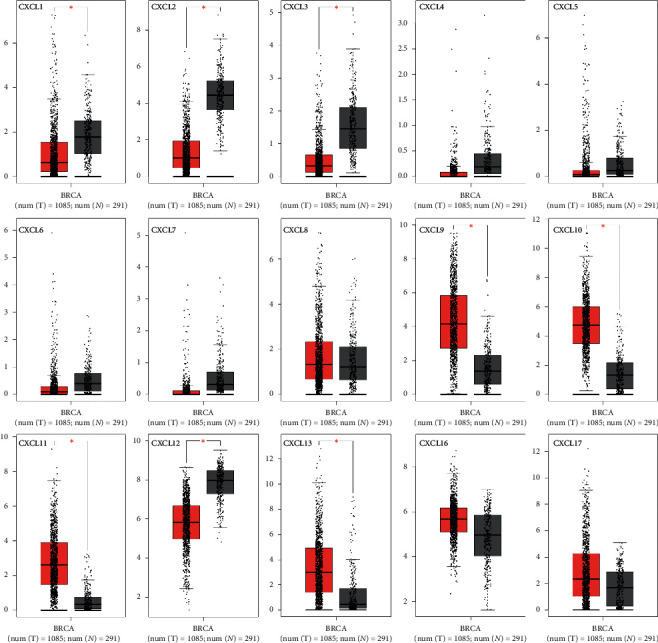
The expression of CXCLs in breast cancer (GEPIA). ^*∗*^*p* < 0.05 (red for tumor, black for normal).

Fifteen different CXCLs family members have been identified in *Homo sapiens*. In order to explore the potential prognostic and therapeutic value of different CXCLs members in breast cancer patients, we compared the transcriptional levels of CXCLs by using Oncomine database and GEPIA. As shown in Figure 1, Oncomine database revealed that mRNA expressions of CXCL9/10/11 were significantly higher in human breast cancer than in normal tissues in multiple datasets and transcription levels of CXCL1/2/4/5/7 were prominently low. Then, GEPIA was applied to measure expression levels based on TCGA and the GTEx database. Expressions on box plots are presented in Figure 2. The results indicated that the expression levels of CXCL9, CXCL10, CXCL11, and CXCL13 were marked higher in breast cancer tissues than in normal tissues, while the expression levels of CXCL1, CXCL2, CXCL3, and CXCL12 were lower inversely.

### 3.2. Prognostic Value of mRNA Expression of CXCLs in Breast Cancer Patients

**Figure 3:**
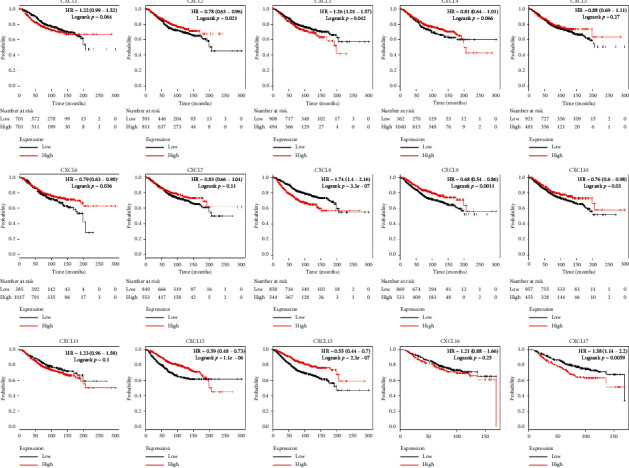
The prognostic value of mRNA level of CXCLs family members in breast cancer patients in the overall survival (OS) curve (Kaplan–Meier Plotter).

Furthermore, we explored the prognostic values of mRNA expression of CXCLs in breast cancer patients by using Kaplan–Meier survival function. The Kaplan–Meier curve and log rank test analyses revealed that the high CXCL2, CXCL6, CXCL9, CXCL10, CXCL12, and CXCL13 mRNA levels were significantly associated with higher overall survival (OS) (*p* < 0.05) (Figure 3) of breast cancer patients, while low mRNA levels of CXCL3, CXCL8, and CXCL17 were predicted to have higher OS. Presented in Supplementary Figures S1 and S2, we also found that high CXCL1/2/3/12/16 expressions were significantly correlated with better RFS (relapse-free survival) in ER-positive breast cancer, and high CXCL8/9/13 expressions were significantly correlated with better RFS in ER-negative breast cancer. It is interesting to note that high CXCL10/11 expressions were significantly correlated with poor RFS in ER-positive breast cancer while better RFS in ER-negative breast cancer.

### 3.3. Association of mRNA Expression of CXCLs with Known Prognostic Factors of Breast Cancer Patients

**Figure 4:**
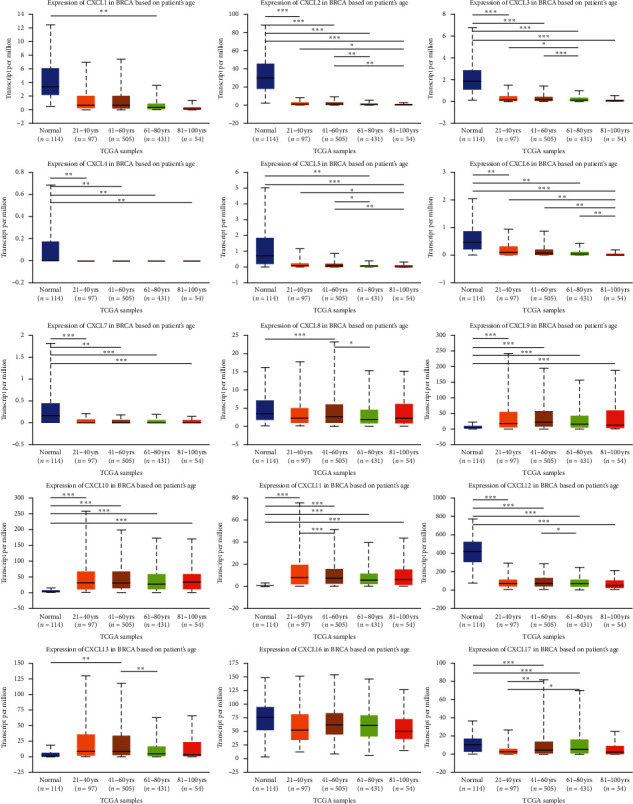
Correlation between mRNA expression of distinct CXCLs family members and age of breast invasive carcinoma patients (UALCAN). ^*∗*^*p* < 0.05, ^*∗∗*^*p* < 0.01, ^*∗∗∗*^*p* < 0.001.

**Figure 5:**
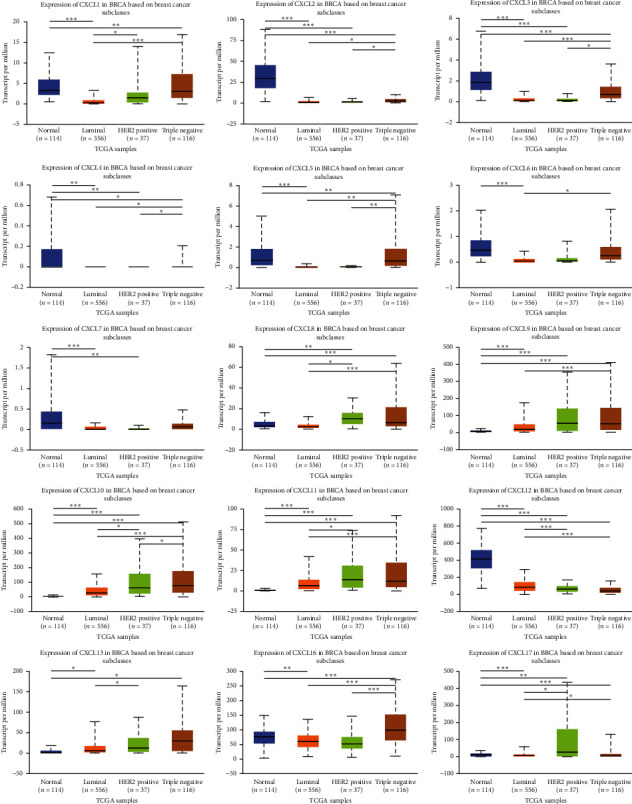
Correlation between mRNA expression of distinct CXCLs family members and major subclasses of breast invasive carcinoma patients (UALCAN). ^*∗*^*p* < 0.05, ^*∗∗*^*p* < 0.01, ^*∗∗∗*^*p* < 0.001.

**Figure 6:**
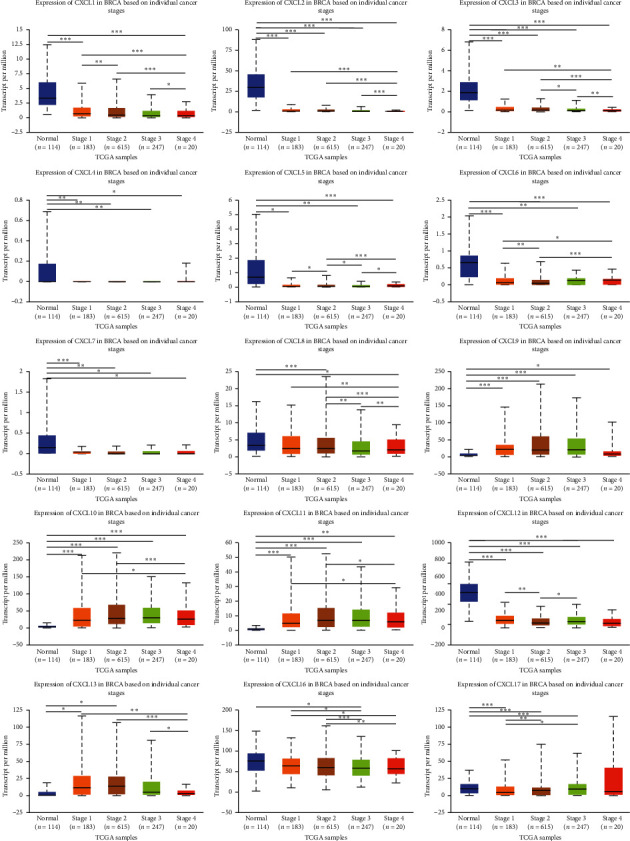
Correlation between mRNA expression of distinct CXCLs family members and individual cancer stages of breast invasive carcinoma patients (UALCAN). ^*∗*^*p* < 0.05, ^*∗∗*^*p* < 0.01, ^*∗∗∗*^*p* < 0.001.

**Figure 7:**
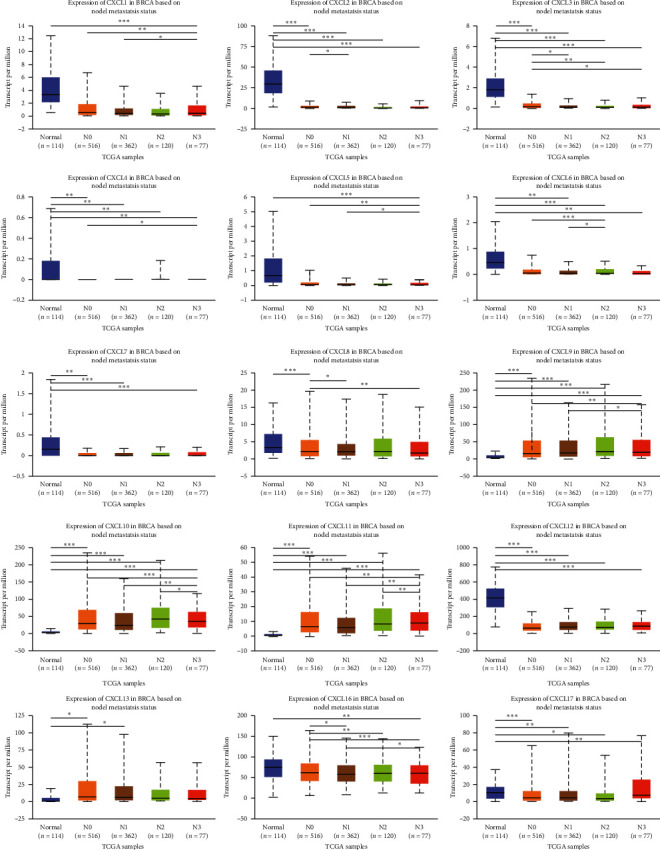
Correlation between mRNA expression of distinct CXCLs family members and nodal metastasis status of breast invasive carcinoma patients (UALCAN). ^*∗*^*p* < 0.05, ^*∗∗*^*p* < 0.01, ^*∗∗∗*^*p* < 0.001.

Correlations between the mRNA levels of CXCLs and known prognostic factors of breast cancer patients were further analyzed in UALCAN. As presented in Figure 4, CXCLs are differently expressed in different age groups. And we can draw conclusions from Figure 5 that CXCL1/2/3/5/6/7 are found significantly expressed in Luminal, CXCL8/11/17 in HER2 positive, and CXCL9/10/12/13/16 in triple negative breast cancer. Also, the transcriptional expressions of CXCLs family members were correlated with patients' individual cancer stages and nodal metastasis status, especially for CXCL2, CXCL3, CXCL4, CXCL7, CXCL9, CXCL10, CXCL11, and CXCL12 (shown in Figures 6 and 7).

### 3.4. Correlation between CXCLs mRNA Expression and Immune Cells by TIMER

**Figure 8:**
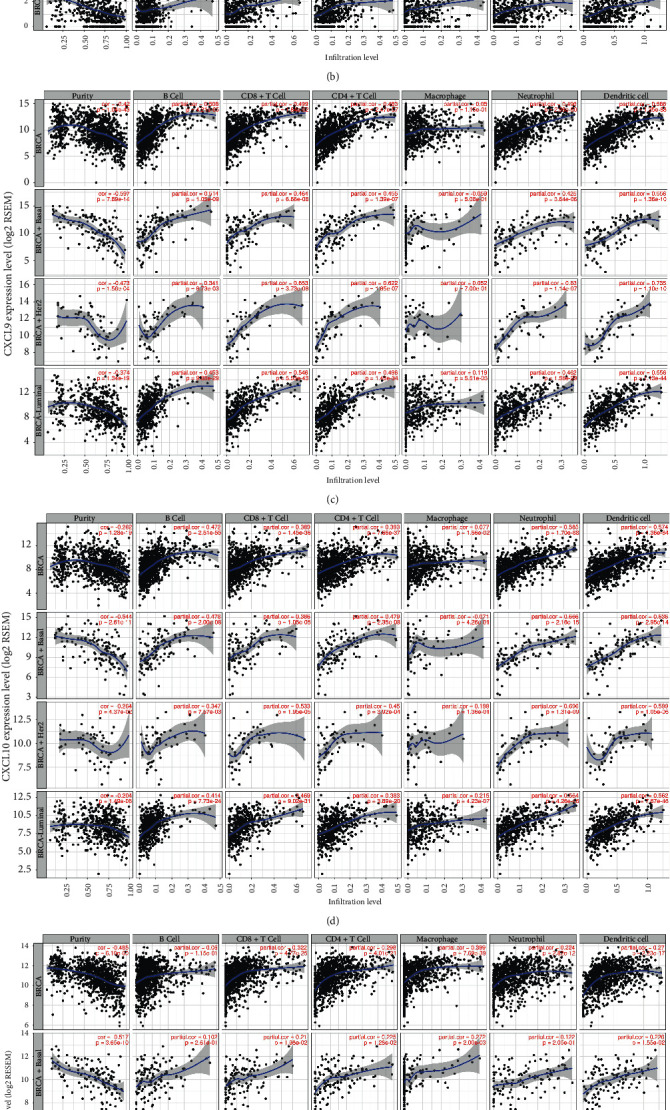
Association of CXCLs (CXCL2, CXCL3, CXCL9, CXCL10, CXCL12, and CXCL3) expression with immune infiltration level in breast cancer patients (TIMER).

Drawn from the above results, CXCL2, CXCL3, CXCL9, CXCL10, and CXCL12 together with CXCL13 were all significantly expressed and associated with the overall survival in breast cancer patients. The expressions were also connected to tumor stages. We then performed correlation analysis between the six CXCLs family members and tumor immune cells by TIMER in patients with breast cancer (BRCA) and different subtypes (BRCA-Basal, BRCA-Her2, and BRCA-Luminal) to assess the effectiveness of immunotherapy. CXCL9 expression level had obviously positive correlations with infiltrating levels of B cells (*r* = 0.508, *p*=4.21*e* − 65), CD8+ T cells (*r* = 0.499, *p*=1.84*e* − 62), CD4+ T cells (*r* = 0.483, *p*=2.47*e* − 57), neutrophils (*r* = 0.496, *p*=2.98*e* − 60), and dendritic cells (*r* = 0.586, *p*=1.35*e* − 88) in BRCA as presented in Figure 8, identified to be a significantly favorable factor in BRCA-Basal, BRCA-Her2, and BRCA-Luminal. CXCL10 showed similar correlations of B cells (*r* = 0.472, *p*=2.51*e* − 55), CD8+ T cells (*r* = 0.389, *p*=1.45*e* − 36), CD4+ T cells (*r* = 0.393, *p*=7.66*e* − 37), neutrophils (*r* = 0.585, *p*=1.70*e* − 88), and dendritic cells (*r* = 0.574, *p*=1.36*e* − 84). In addition, CXCL13 expression had significant correlations with immune cells irrespective of subgroups. It is interesting to note that the mRNA expression of CXCL12 was meaningful associated with macrophages (*r* = 0.399, *p*=7.69*e* − 39) in BRCA, especially in BRCA-Her2 (*r* = 0.445, *p*=4.71*e* − 04) and BRCA-Luminal (*r* = 0.417, *p*=2.93*e* − 24).

### 3.5. Function Enrichment Analysis of Favorable CXCLs

**Figure 9:**
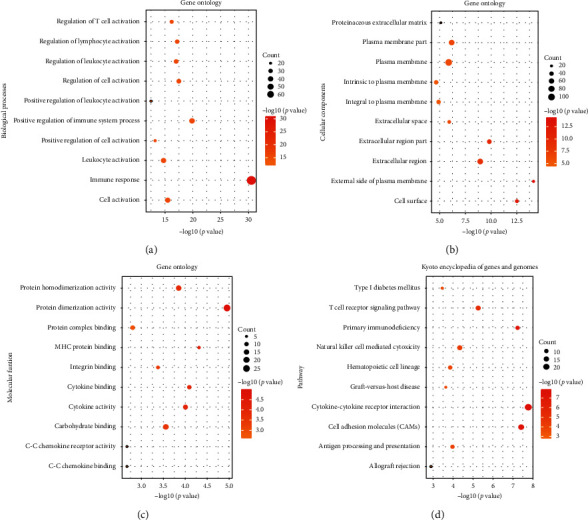
GO functional enrichment analysis on CXCL9, CXCL10, CXCL12, and CXCL13 as well as their 100 frequently altered neighbor genes in breast cancer patients. (a) Biological process. (b) Cellular components. (c) Molecular functions. (d) KEGG pathway analysis on CXCL9, CXCL10, CXCL12, and CXCL13 as well as their 100 frequently altered neighbor genes in breast cancer patients.

We first obtained the top 100 similar expression protein-coding genes with CXCL9, CXCL10, CXCL12, and CXCL13, respectively, by similar genes module in GEPIA. We then performed GO and KEGG analysis by DAVID online (https://david.ncifcrf.gov/) and R project. Presented in Figure 9, biological processes such as immune response, positive regulation of immune system process, and cell activation were remarkably regulated by the four CXCLs in breast cancer patients. Cellular components were mainly focused on plasma membrane, extracellular region, and plasma membrane part. Protein dimerization activity, protein homodimerization activity, and carbohydrate binding function correlated closely with the mRNA expression of CXCL9, CXCL10, CXCL12, and CXCL13. Among the top 10 KEGG analysis, cytokine-cytokine receptor interaction, cell adhesion molecules (CAMs), T cell receptor signaling pathway, and natural killer cell mediated cytotoxicity held the majority.

## 4. Discussion

CXCLs were recently discovered to play important roles in various cancer types, including colorectal cancer, pancreatic cancer, lung cancer, and hepatocellular carcinoma [4, 6, 20, 21]. However, the prognostic value and biological function of CXCLs in breast cancer have not been well illustrated. CXCL9/10/12/13 are all angiostatic ELR-members. CXCL9 and CXCL10 both bind to CXCR3, which is associated with T cell function [22]. CXCL9 has been identified as a candidate biomarker in breast cancer. Denkert's study showed that high CXCL9 expression conferred a significantly increased pathologic complete response rate (pCR) in breast cancer patients who received neoadjuvant anthracycline/taxane‐based chemotherapy [23]. However, CXCL9 contributes to not only tumor inhibition but also tumor promotion. Ejaeidi et al. showed that the levels of CXCL9 and CXCL10 were markedly high in 40 HR- (hormone receptor-) positive metastatic breast cancer patients when compared to HR-negative patients and healthy controls in plasma. CXCL10, also known as IFN-g-inducible Protein 10, plays an important role in promoting the homing of immune cells that mediates the subsequent death of cancer cells in breast cancer. Our study showed high CXCL9/10 expression significantly correlated with favorable survival outcome in breast cancer. Combined with the results in ovarian cancer [25], we surmise that CXCL9 and CXCL10 exert tumor-suppressive function by TIL recruitment through the JAK/STAT and NF-*κ*B pathways. What is more, studies also revealed that PARP inhibitor-Olaparib induced T cell recruitment is mediated through activation of the cGAS/STING pathway, whose major effector, IRF3, has been reported to regulate expression of CXCL10 [26]. CXCL13, originally named B cell attracting chemokine 1, is also identified to be correlated with CXCL9 (rho = 0.52, *p* < 0.001) in early breast cancer [27]. Interestingly, the combined expression was also associated with TIL. CXCL13 has been reported to contribute to cancer progression in breast cancer by recruiting B lymphocytes into tumor microenvironment [4, 28]. Zeng et al. also found the correlation of CXCL9/10/13 with immune infiltration in renal cell carcinoma, which was similar to the results in our study [5]. CXCL12 binds to CXCR4 and CXCR7 to promote cancer progression and promotes chemoresistance invasion and migration by activating a number of intracellular signaling molecules, including Akt, EGFR, mTOR, NF-*κ*B, and Src [29, 30]. A recent study indicates enhanced infiltration of T lymphocytes and natural killer cells in the tumor microenvironment by blocking CXCL12 [31].

Several limitations should also be mentioned in our study. Firstly, multicenter, large-scale prospective studies with long-term follow-up are still urgently needed, for conclusions drawn from different databases may be inconsistent and not wholly reliable Secondly, it is noted that chemokines play a dual role in tumor progression. However, variant mechanisms have not been well clarified in different cancers. For example, it is worth a deeper look at the complicated relation among CXCLs and the NF-*κ*B signaling pathway. In conclusion, our study found additional evidence that CXCL9/10/12/13 could be used as potential prognosis biomarkers in breast cancer.

## 5. Conclusions

In this study, we analyzed the expression of 15 different CXCLs family members in breast cancer and adjacent tissues or normal tissues and assessed the relationship between CXCLs expression and overall survival, as well as tumor staging. Our findings indicated that high CXCL2/9/10/12/13 expression and low CXCL3 expression were associated with favorable OS in breast cancer patients. All of them are significantly linked with patients' known prognostic factors, such as patient's age, major subclasses, individual cancer stages, and nodal metastasis status. Tumor-infiltrating lymphocytes (TILs) are recently found predicting neoadjuvant chemotherapy response in different breast cancer subtypes [32]. It is considered that immune cells have a large impact on the tumor immune network and tumor development. We then utilized TIMER to perform the relationships between CXCL2/3/9/10/12/13 expression and abundance of immune infiltrates, as well as conducting the GO and KEGG analysis. The results suggested that CXCL9/10/13 expressions were significantly associated with infiltrating levels of B cells, CD8+ T cells, CD4+ T cells, neutrophils, and DCs in breast cancer, while CXCL12 was conversely correlated with macrophages. In recent years, with the breakthroughs in the research of immunological checkpoint, patients have received unprecedented long-lasting anti-tumor response, where immune microenvironment, tumor-infiltrating cells, and immune biomarkers play important roles. Our study indicated that the mRNA expressions of CXCL9/10/12/13 are correlated with immune cells. Therefore, we speculated that they could be used to predict the effectiveness of immune blockade therapy in breast cancer patients.

## Data Availability

The datasets generated and analyzed during the current study are available in the Oncomine database (http://www.oncomine.org), GEPIA (http://gepia.cancer-pku.cn/), UALCAN (http://ualcan.path.uab.edu), Kaplan–Meier Plotter (http://www.kmplot.com), TIMER (https://cistrome.shinyapps.io/timer/), and DAVID (https://david.ncifcrf.gov/home.jsp).

## Supplementary Materials

Supplementary Materials Figure S1: the prognostic value of mRNA level of CXCLs family members in ER-positive breast cancer patients in the relapse-free survival (RFS) curve (Kaplan–Meier Plotter). Figure S2: the prognostic value of mRNA level of CXCLs family members in ER-negative breast cancer patients in the relapse-free survival (RFS) curve (Kaplan–Meier Plotter).
