# Supplementary material for: Transcriptional Expressions of CXCL9/10/12/13 as Prognosis Factors in Breast Cancer
Source: J Oncol. 2020 Sep 9;2020:4270957. doi: 10.1155/2020/4270957 (PMC7499319; doi:10.1155/2020/4270957)
Supplement: Supplementary Materials — Figure S1: the prognostic value of mRNA level of CXCLs family members in ER-positive breast cancer patients in the relapse-free survival (RFS) curve (Kaplan–Meier Plotter). Figure S2: the prognostic value of mRNA level of CXCLs family members in ER-negative breast cancer patients in the relapse-free survival (RFS) curve (Kaplan–Meier Plotter). [file 4270957.f1.zip › 4270957.f1/S2.pdf]

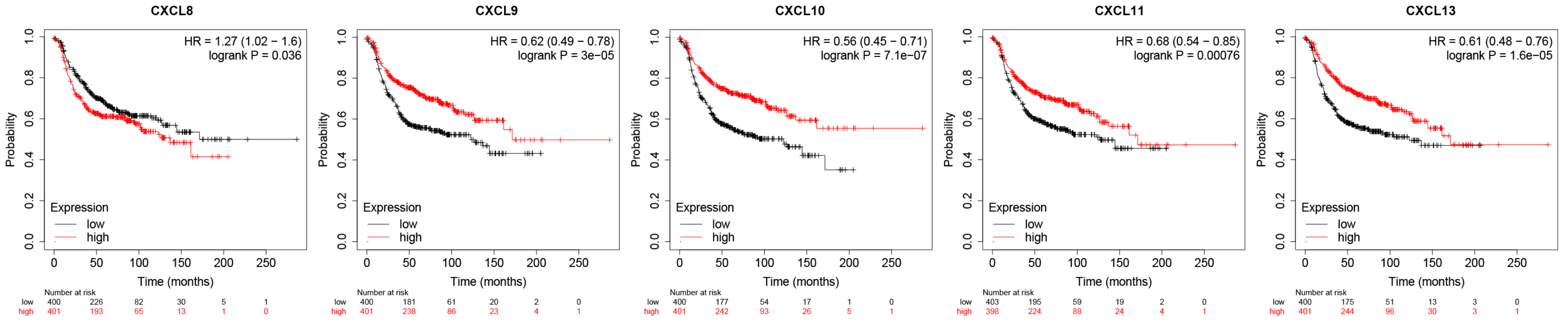

CXCL8
HR = 1.27 (1.02 − 1.6)
logrank P = 0.036
Probability
Time (months)
Expression
low
high
Number at risk
low 400 226 82 30 5 1
high 401 193 65 13 1 0
CXCL9
HR = 0.62 (0.49 − 0.78)
logrank P = 3e−05
Probability
Time (months)
Expression
low
high
Number at risk
low 400 181 61 20 2 0
high 401 238 86 23 4 1
CXCL10
HR = 0.56 (0.45 − 0.71)
logrank P = 7.1e−07
Probability
Time (months)
Expression
low
high
Number at risk
low 400 177 54 17 1 0
high 401 242 93 26 5 1
CXCL11
HR = 0.68 (0.54 − 0.85)
logrank P = 0.00076
Probability
Time (months)
Expression
low
high
Number at risk
low 403 195 59 19 2 0
high 398 224 88 24 4 1
CXCL13
HR = 0.61 (0.48 − 0.76)
logrank P = 1.6e−05
Probability
Time (months)
Expression
low
high
Number at risk
low 400 175 51 13 3 0
high 401 244 96 30 3 1
