# Supplementary figures and images for: Transcriptional Expressions of CXCL9/10/12/13 as Prognosis Factors in Breast Cancer
Source: J Oncol. 2020 Sep 9;2020:4270957. doi: 10.1155/2020/4270957 (PMC7499319; doi:10.1155/2020/4270957)

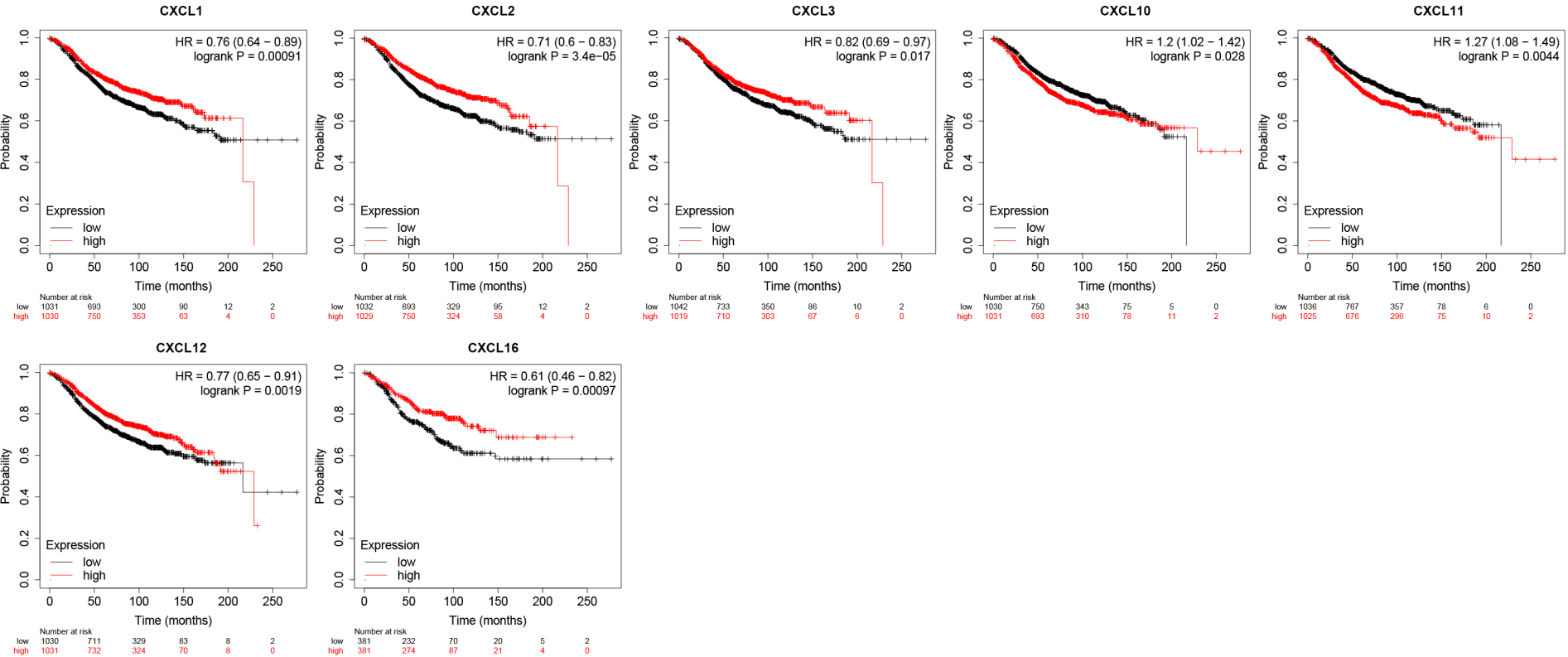

Supplement: Supplementary Materials — Figure S1: the prognostic value of mRNA level of CXCLs family members in ER-positive breast cancer patients in the relapse-free survival (RFS) curve (Kaplan–Meier Plotter). Figure S2: the prognostic value of mRNA level of CXCLs family members in ER-negative breast cancer patients in the relapse-free survival (RFS) curve (Kaplan–Meier Plotter). [file 4270957.f1.zip › 4270957.f1/S1.pdf]
